# Editorial: The methane moment - Cross-boundary significance of methanogens: Preface

**DOI:** 10.3389/fmicb.2022.1055494

**Published:** 2022-11-14

**Authors:** Zhe Lyu, Amelia-Elena Rotaru, Mark Pimentel, Cui-Jing Zhang, Simon K.-M. R. Rittmann

**Affiliations:** ^1^Department of Molecular Virology and Microbiology, Baylor College of Medicine, Houston, TX, United States; ^2^Alkek Center for Metagenomics and Microbiome Research, Baylor College of Medicine, Houston, TX, United States; ^3^Nordic Center for Earth Evolution (NORDCEE), University of Southern Denmark, Odense, Denmark; ^4^Medically Associated Science and Technology (MAST) Program, Cedars-Sinai, Los Angeles, CA, United States; ^5^Archaeal Biology Center, Institute for Advanced Study, Shenzhen University, Shenzhen, China; ^6^Archaea Physiology & Biotechnology Group, Department of Functional and Evolutionary Ecology, Universität Wien, Vienna, Austria; ^7^Arkeon GmbH, Tulln a.d. Donau, Austria

**Keywords:** methanogenesis, microbiome, host, environment, agriculture, energy, health, biotechnology

## Introduction

Methanogens are anaerobic methane-producing archaea that derive energy from methanogenesis, a biological process responsible for most global methane emissions. Because methane is a potent greenhouse gas and a high-energy fuel, methanogens could help solve the dual challenge humanity is facing—climate change and energy shortage (Buan, 2018). On the one hand, mitigating methane emissions is a high priority in tackling global warming and climate change. On the other hand, promoting methane production in well-controlled environments such as waste digesters convert waste into high purity methane as a sustainable biofuel.

There are also good reasons to believe that the significance of methanogens would extend beyond climate and energy. For example, methanogens were one of the earliest life forms on Earth, they have evolved enormous diversity for ~3.5 billion years (Wolfe and Fournier, 2018), they possess many essential genes uniquely shared between archaea and eukaryotes (Lyu and Whitman, 2017), and they are now collectively distributed in a wide range of ecosystems—on the land, in the oceans, across extreme environments (Liu and Whitman, 2008), closely associated with humans, animals, and plants (Borrel et al., 2020), and even adapted to oxic niches (Lyu and Lu, 2018). A good understanding and translation of their functions across the biosphere will unravel the untapped cross-boundary significance of methanogens relevant to the environment, energy, agriculture, biotechnology, and health and disease of humans, animals, and plants.

Therefore, we have formed a guest editorial team with diverse expertise in microbiology, environmental science, biotechnology, and medicine to bring forward this new Frontiers Research Topic “*The Methane Moment—Cross-boundary Significance of Methanogens*” (Lyu et al., 2022). To showcase methanogen-relevant research across multiple disciplines, this topic is cross listed with 3 Frontiers journals and 6 sections. To facilitate the discussion of this topic, our editorial team presents here a preface article envisaging, within our expertise, the significance of methanogens in diverse settings.

### Evolution of methanogens and methanogenesis

While the origin of methanogens and their ancestral pathway of methanogenesis is debatable, modern methanogens operate five unique pathways: CO_2_-reducing, aceticlastic, methylotrophic (Lyu et al., 2018b), methoxydotrophic (Mayumi et al., 2016) and alkylotrophic (Zhou et al., 2022). All the pathways use a methyl-coenzyme M reductase complex (MCR) to catalyze the final step of methanogenesis. MCR homologs are also shown to be involved in anaerobic methanotrophy and alkane metabolism (Laso-Perez et al., 2016; Borrel et al., 2019). Previously, it was reported that methanogens were classified into two distantly related groups within the phylum Euryarchaeota (Bapteste et al., 2005; Borrel et al., 2016). In recent years, genomic and sequencing analysis has proposed many novel methanogens outside Euryarchaeota, expanding the diversity of methanogens and methanogen-like archaea (Baker et al., 2020). For example, Korarchaeota, Thaumarchaeota, Verstraetearchaeota, and Nezhaarchaeota, also contain MCRs, suggesting that they have the potential of methanogenesis (Wang et al., 2019). Bathyarchaeota, Hadesarchaea, and Helarchaeota contain alkyl–coenzyme M reductase complex (ACR), enzymes that are similar to MCR but activate alkanes instead (Seitz et al., 2019).

The findings of MCR and ACR in non-Euryarchaeota challenged the hypothesis that methanogenesis originated from Euryarchaeota, suggesting a more complex evolutionary history of methanogens (Garcia et al., 2022). Some studies suggested that the hydrogen-dependent CO_2_-reducing (hydrogenotrophic) methanogenesis may be the ancestral form of biological methane production and methanogens evolved from hydrogenotrophic to methylotrophic methanogenesis (Berghuis et al., 2019). Others suggested that hydrogen-dependent methylotrophic methanogenesis was the ancestral form and hydrogenotrophic methanogenesis developed later by phylogenetic analysis of methanogens based on concatenated ribosomal proteins and on functional proteins (Wang et al., 2021). Further studies on the origin, evolution, and ecophysiology of methanogens will be instrumental in understanding their adaptation across the biosphere and their cross-boundary significance thereof.

### Significance in the global carbon cycle

As part of the Earth's ‘biogeochemical engine', methanogens transform annually 2% of the ~70 Gt global net primary production carbon into ~1 Gt of methane, of which ~60% is oxidized by methane oxidizers. The remaining ~0.4 Gt escapes into the atmosphere, accounting for ~70% of the global methane emission (Thauer et al., 2010). Notably, this well-established model does not consider that biological methane emission can also occur in non-methanogens which may encompass all living organisms including plants, fungi, algae, bacteria, archaea, and human cells (Günthel et al., 2019; Bižić et al., 2020; Ernst et al., 2022). Non-methanogens do not produce methane via methanogenesis where substrates are stoichiometrically converted into methane for energy conservation. Instead, their methane appears to be a metabolic byproduct likely derived from methyl radicals induced by reactive oxygen species under oxic conditions. Consequently, the observed methane yield in non-methanogens is extremely unstable and varies in the range of sub-attomole to micromole per gram of dry cellular weight (Ernst et al., 2022), dwarfed by typical methanogens yielding at the mole level (Thauer et al., 2008). Moreover, methanogens predominate all the major methane-emitting habitats such as ruminants and rice fields (see below). Therefore, while it is of significance to develop a quantitative global model for the elusive methane emissions from non-methanogens, methanogens remain the most potent biological methane producer and the top contributor to the global methane emission.

Abiotic sources such as mining and combustion of fossil fuel and biomass burning contribute to ~30% of the global methane emission (Conrad, 2009; Rosentreter et al., 2021). Since the industrial revolution, the atmospheric methane has almost tripled from ~700 to an alarming ~1900 ppb, contributing substantially to global warming and climate change (Earth Org, 2022). The increase in abiotic emission is almost exclusively human-induced, while a large share of that increase in biological emission is also anthropogenic (Conrad, 2009). About 40% of the biological methane come from methanogens in the ruminants and rice fields for producing meat, milk, and rice, and another ~16% from methanogens in landfills and sewage treatment facilities (Lyu et al., 2018b). As the world population continues to grow, food consumption and waste disposal will inevitably increase, fueling more methane emissions. This creates a nexus of heated conflicts between global warming, food and agriculture security, and waste management. To add insult to injury, biological emission from at least certain natural sources such as tropical wetlands may have entered a positive feedback loop (Voosen, 2022). In these wetlands, elevated methane emission makes climate warmer and wetter which in turn fuels more methane emission. This is of immense concern, as wetlands are a top habitat for methanogens and the single largest natural source of methane emission, responsible for emitting ~0.15 Gt of methane annually (Lyu et al., 2018b; Rosentreter et al., 2021).

### Significance in environmental microbiome

Methanogens proliferate in natural and engineered habitats limited in typical electron acceptors (O_2_, nitrate, or sulfate), such as deep subsurface environments (Underwood et al., 2022), intestinal tracts of animals and insects (Borrel et al., 2020), and anaerobic digestors for residential and industrial waste treatment (Vítězová et al., 2020; De Bernardini et al., 2022). Here, methanogens act as a terminal electron sink driving anaerobic oxidation of organic matter to completion. Specifically, methanogens syntrophically couple their reductive metabolism with bacterial partners' oxidative metabolism via indirect or direct electron transfers facilitated by H_2_/formate (Schink, 1997), conductive mineral grains like iron-oxides or activated carbon (Rotaru et al., 2018), or electron-carrying cell surface molecules such as multiheme c-type cytochromes (Rotaru et al., 2021).

A general assumption is that the thermodynamically more efficient respiratory bacteria such as nitrate and sulfate reducers would competitively displace methanogens in habitats where typical electron acceptors are abundant. However, that is not always the case. For example, in oxic and sulfate-rich seagrass meadows, methylotrophic methanogens occupied an unconventional niche via demethylation of compounds like betaine, an osmolyte of seagrasses (Schorn et al., 2022). This unconventional niche may even harbor novel methanogens or methanogen-like archaea, evidenced by the presence of *Ca*. Helarchaeota metagenomes encoding the *mcrA* (Schorn et al., 2022). Other unconventional niches include steel structures suffering corrosion (Lahme et al., 2021), and electrode materials for bio-electricity generation (Aryal et al., 2022). These examples highlight the robustness of methanogens in adapting to a wide range of habitats. Regardless of the habitats, a key to the proliferation of methanogens is to obtain electrons from their surrounding microbiome. Further studies on this electron exchange process will contribute to a functional assessment of the overall electron flow in environmental microbiomes. This has implications for methane mitigation, biofuel production, and ecosystem stability.

### Significance in human health and beyond

Methanogens in human health and disease have a complicated story. While detection of gut-derived methane in human breath was first studied in the 1970's (Bond et al., 1971), it was initially believed that methanogens and methane production had no physiological consequence. However, it is now known that methanogens colonize the gastrointestinal tract commonly but when they do so more prominently, they can be associated with human disease. The strongest relationship is with constipation (Triantafyllou et al., 2014). The presence of methane is not only associated with constipation, it is proportional to the amount of methane and studies of intestinal physiology point to methane as the cause and perhaps even as a potential gasotransmitter (Pimentel et al., 2006; Wang, 2014). Studies go on to identify the main human gut methanogen as *Methanobrevibacter* spp. (Kim et al., 2012), and other notable gut methanogens also belong to *Methanosphaera* spp. and *Methanomassiliicoccales* (Hoegenauer et al., 2022).

While typically associated with constipation, gut methanogens and methane is linked to other conditions including obesity. In an elegant study using germ free animals, methanogens promoted obesity when animals were co-colonized with *Bacteroides thetaiotamicron* demonstrating that methanogens are dependent on syntrophic bacteria (Samuel et al., 2007). Work in obesity suggests that methanogens are associated with higher body-mass-index in obesity and their presence may also predict a less ideal outcome for weight loss after bariatric surgery (Basseri et al., 2012; Mathur et al., 2016). Beyond the gut, oral methanogens are strongly associated with polymicrobial oral infections such as periodontitis (Lepp et al., 2004; Horz and Conrads, 2011), while the gut methanogen *Methanobrevibacter smithii* could produce 2-hydroxypyridine that might drive Parkinson's disease pathogenesis (Wilmes et al., 2022). It is clear from these examples that it is important to understand the roles of methanogens at least in the context of nutrition, constipation, polymicrobial infection, and gut-brain axis. Furthermore, basic principles established for human-associated methanogens would have implications in the poorly studied animal- and plant-associated methanogens. Together, studies of these host-associated methanogens will help to elucidate their roles in the health and disease of humans, animals, and plants.

### Significance in biotechnology

Besides their commercial applications in waste treatment and biogas production, methanogens emerge also as cell factories for sustainable biomanufacturing, owing to their intriguing ecophysiological characteristics. Notable examples include their high gas or hydrostatic pressure tolerance, ranging from 300 kPa to 400 MPa (Ver Eecke et al., 2013; Taubner et al., 2018; Pappenreiter et al., 2019); their wide range of growth temperature, from approx. −4 to 122°C (Taubner et al., 2015); and their abilities to reduce CO_2_ with diverse gaseous and volatile compounds such as H_2_, CO, formate, ethanol, and secondary alcohols (Kurth et al., 2020). Moreover, certain methanogens are also fast growing (Abdel Azim et al., 2017; Palabikyan et al.) and genetically tractable (Mondorf et al., 2012; Nayak and Metcalf, 2017; Susanti et al., 2019; Lyu et al., 2020; Fink et al., 2021; Bao et al., 2022; Li et al., 2022).

With these features, methanogens are poised to help drive the next biotechnological boom through a gas fermentation bioprocess. Of special interests to this process are CO_2_-reducing microbes, which can be regarded as a carbon-negative cell factory that enables carbon fixation, bioenergy transition, and production of high-value bioproducts (Müller, 2019; Pfeifer et al., 2021; Liew et al., 2022). As a proof of concept, methanogens capable of CO_2_-reduction have already been engineered to produce geraniol (Lyu et al., 2016), isoprene (Aldridge et al., 2021), acetate (Schone et al., 2022), enzymes (Lyu et al., 2018a; Akinyemi et al., 2021), and bioplastics (Thevasundaram et al., 2022) under laboratory conditions. Additionally, several high performance and high pressure gas fermenting methanogen cell factories have been identified under bioreactor conditions (Mauerhofer et al., 2018, 2021). Because the biomass of methanogens is already rich in valuable compounds such as ether lipids (Baumann et al., 2018, 2022), carboxylic acids, and complex coenzymes (Lyu and Whitman, 2019), coupling synthetic biology with bioprocess development holds high potential for advancing methanogens into an economically feasible platform for green biomanufacturing.

## Discussion

Although invisible to the naked eye, many have acknowledged the significant influence of methanogens and their surrounding microbiome on humanity's fate. This is evidenced by the Global Methane Pledge endorsed by 120 nations aiming for a 30% cut in methane emissions by 2030 (The White House, 2021). However, beyond methanogenesis and methane reduction, more research is needed to understand the functional roles of methanogens in both free-living and host-associated microbiomes. Bioengineering of and bioprocess development for methanogens are also of high importance, which echoes the unprecedented investments in low-carbon biomanufacturing (The White House, 2022). Ultimately, the convergence of these research areas would lead to a more sustainable future by not only mitigating global warming and climate change but tapping the potential of methanogens in environmental, agricultural, industrial, and medical biotechnology.

## Author contributions

ZL coordinated this collaboration and drafted the sections—Introduction, Significance in the global carbon cycle, Discussion, and edited the manuscript. A-ER drafted the section—Significance in environmental microbiome. MP drafted the section—Significance in human health and beyond. C-JZ drafted the section—Evolution of methanogens and methanogenesis. SK-MRR drafted the section—Significance in biotechnology. All authors contributed to the article and approved the submitted version.

## Funding

ZL is supported by the NIH/NIAID grant U19AI157981 and seed funds provided by the Baylor College of Medicine. A-ER is supported by the NovoNordisk Foundation grant NNF21OC0067353, the Danish Research Council grant DFF 1026-00159 and the European Research Council ERC-CoG grant 101045149. C-JZ is supported by the National Natural Science Foundation of China (no. 42007217). Research in the group of SK-MRR at the Universtität Wien is supported by the Forschungsförderungsgesellschaft (FFG) grant 888509, project FlaeXMethan.

## Conflict of interest

Author SK-MRR is co-founder of Arkeon GmbH. Author MP is a consultant for Bausch Health, Ferring Pharmaceuticals Inc., Salvo Health, and Vivante Health Inc.

The remaining authors declare that the research was conducted in the absence of any commercial or financial relationships that could be construed as a potential conflict of interest.

## Publisher's note

All claims expressed in this article are solely those of the authors and do not necessarily represent those of their affiliated organizations, or those of the publisher, the editors and the reviewers. Any product that may be evaluated in this article, or claim that may be made by its manufacturer, is not guaranteed or endorsed by the publisher.
